# Supplementary material for: “Some believe those who say they can cure it” perceived barriers to antiretroviral therapy for children living with HIV/AIDS: Qualitative exploration of caregivers experiences in tamale metropolis
Source: PLoS One. 2022 Oct 4;17(10):e0275529. doi: 10.1371/journal.pone.0275529 (PMC9531795; doi:10.1371/journal.pone.0275529)
Supplement: S1 File — (DOCX) [file pone.0275529.s001.docx]

## Interview Guide

**INTERVIEW GUIDE**

**Experiences of Caregivers of Children Living with HIV and AIDS in Tamale Metropolis, Northern-Ghana**

**Section: A**

**Biographic Data**

**Caregiver Information**

**Hospital: ____________ Date: ________________ Time: ___________Hours GMT**

**Age: ____________years Sex: ______________ Religion: ____________**

**Tribe: _______________________________ Level of education: ________________**

**Number of children or people he/she is caring for: ______________**

**Relationship with child: ___________________ Head of the family: _________________**

**Child Information**

**Age: ____________________________ How long has been on ART: _______________**

**Level of education: ________________ General Health: ______________________________**

**Participant Signature/ thumbprint: ______________ Date: _______________________**

**Name of Research Member: __________________ Signature: ____________________**

**Section: B**

**Make participant comfortable and greet according to culture**

**Introduce self**

**Reassure participant and re- detail the purpose of the interview**

**Can you tell me about yourself?**

Probing about

- other roles aside being a care giver
- Family: family composition, economic strength
- Type of family (extended or nuclear)
- Family mechanism, head, breadwinner, decision making
- Family relationships/family cohesion
- Who takes decisions about health in the family, health seeking behavior?

**Can you tell me about your child?**

- How long have you been caring for your child?
- Since when did you notice your child was sick was sick
- How, person, place, time, date and under what circumstances.

**Can you tell me about how you seek care and support?**

- What happen whenever your child is sick is sick
- What support do you get and from whom?
- Note from: family, friends, neighbors, community leaders, traditional leaders, spiritual leaders (probe for stories of help and services)
- What do you think of the support they give?

**What are the roles and activities they take in the care of your child?**

- Probe detail time and routines of caring for your child: food, shopping, medications among others

**Emotional, spiritual and physical support/coping/relax**

- What are some of the ways you are able to cope with your child’s condition and stress that comes with caring for your child and other roles.

**Disclosure**

- Tell me who knows about your child sickness
- Tell me whom you wish to also tell and why
- Whom do you not want to tell and why?

**Tell me about the care from the hospital**

- How do you feel about the care given?
- What are your expectations of the care given by hospital, institution, NGO (if any)?

**Can you tell me about home and hospital medications for x?**

Our records show seven out of ten children with your child conditions are not brought to the hospital to take treatment.

- What are some of the reasons that might account for this in your own view?
- What are some of the problems or challenges your child experiences with ART?

**What is the most challenging issue/ day in caring for your child?**

**What kind of discriminations do you encounter due to your child condition?**

**What are your plans for x in the future**?

**What else do you want to tell me?**

**Thank participant**

**Note: After interview provide the care-giver with a bar of soap and a snack/toffee to the child.**
