# Supplementary material for: “Some believe those who say they can cure it” perceived barriers to antiretroviral therapy for children living with HIV/AIDS: Qualitative exploration of caregivers experiences in tamale metropolis
Source: PLoS One. 2022 Oct 4;17(10):e0275529. doi: 10.1371/journal.pone.0275529 (PMC9531795; doi:10.1371/journal.pone.0275529)
Supplement: S2 File — (DOCX) [file pone.0275529.s002.docx]

**TRANSCRIPT**

**Caregiver 1. Tch 001**

**Can you tell me about yourself?**

Ammm...my name is ……. and I live in Tamale, I came here for this thing I came here for medication. I don’t do anything {work} aside from being am a trader but my husband is a customs {officer}, we are four so {your husband and 2 children} which is a nuclear family. In the family, my husband is the breadwinner, head of the family, who makes decisions concerning health. And we do have a good relationship with the extended family when it comes to your daily activities, social life

**Can you tell me about x? Talk about the girl, how you got to know about her condition**

ehhmmaaaaahh I have been caring for her since birth but it is about three years now it was that time that we came to know (she is positive to the virus)

hmmmm they told us from the hospital where she was admitted and they give us eehhh this thing (lab request for retro-screen) to go for lab test when we went and did it. I myself went for the results and I open the results to see and it was positive and I came to the, so we find out from the hospital. And I take it normal, my husband too took it normal because I am also positive so we all took it normal, we all take it normal, we pray to God. I was normal because am also one of them, with the disease. So for me and my husband, we took it as normal, because for us once one of us is surviving with it. So to our child to have it, is normal. We just go back to the hospital, because there!!! This thing the doctor who tell us to go and do the results, when we came he collected the results from us and for the folder he did not write anything in the folder. so when we went to the house we just feel normal with it because there is nothing in the folder that shows that that girl is positive so when we are going to hospital, we just go straight to that doctor. Or anything so we feel normal when we are going to the hospital. (He did not write in the folder) because we are relatives to the doctor and he was, and we almost all the people working in the hospital knows us, so we don’t, my husband told me that ehhmm, no one should know something like that has happened to the child. And to avoid discrimination.

**Can you tell me about how you seek care and support?**

We the support from my husband, and he is the only one who knows that this virus is with us. So Is only my husband who supports / care for us? He is the only one who knows. Ehmmm we have support but not like from (other) people, they are concern and they come to greet but they don’t give us anything. The only support we get from them is comforting, they will only come and talk to you. They don’t give us anything, they don’t give me money. (the support) is enough because you can’t ask someone to do something for you. he will ask you to know the reason why and will ask and you can’t tell the person. Like am sitting now. Asking someone to fetch water for you they will ask you the reason. And if they are to give me money they will want to know the reason. So because of this is enough the support we are getting.

**What are the roles and activities they take in the care of x?**

In the morning after prayer I go to fetch water, then go for selling my things after that I will come back from 1-2(pm) I will give her the medicine for her to take and in at night I will cook and serve give her, her medicine again. That’s all.

**Emotional, spiritual and physical support/coping/relax**

I cope because I see it to be, Eehhhmm I pray to God, I pray to God, always when I pray, I pray to God to give us ehhh this strength to be able to take care of them, hmmmm we buy medicine, if we are not able to go to the hospital, we go and buy medicine

**Disclosure**

Only the doctor and (for the Family), No, only my husband, I never wish to tell anybody, because people are not to be trusted. My husband, my (came to know) knows it when I went to hospital for maternity and there, so when I went there they said their going to do HIV test so when they test me to find out it is positive, they called my husband. I and my husband went for. then I called him. And they test him, for him is negative and they told him reason why their tested him and am positive. That’s where he got to know. I give them the opportunity

**Tell me about the care from the hospital**

I feel normal and feel happy and I don’t feel anything or shy anytime am coming here for the medicine because they take good care of us. The nurses are good to us every month we come. They don’t harsh us, they don’t talk to us like we are nobody, they console us which usually we will not feel bad. Smiling aaaahhh I will give them excellent. because they have done well. i will give them 10/10 because they never do anything that will make us bad. the care they give meet my expectation and I however don’t belong to any organization or institution that is concern with PLWH

**Can you tell me about home and hospital medications for x? perceived barriers to ART and side effects.**

May be sometimes they don’t have time or for the children they may not have someone to come for the medication. For the ones who come for the medication they may be busy and may not get time to come for the medication. And the child like this one can’t come by herself for the medication if not someone who brings her.so that may be the reason for this they are not coming.

No!!! I have not notice such (any side effects) or challenges

**What is the most challenging issue/ day in caring for x?**

Ehhmmmm I don’t have any story because the who takes care of me, takes care of her too. So we don’t have any challenge any serious difficulties in regards to our sickness

**What kind of discriminations do you encounter due to x condition?**

There are no discriminations, because I have not told anybody?

**What are your plans for x in the future**?

My plans for her is to make sure I give her the medications, take care of her and make sure she never lacks anything.

**What else do u want to tell me**

Laughing, I have nothing to say. The only thing I say is they should help us take care of the children with the virus like they should provide the medicine on time, so that they will not miss the medicine. The last time I came and they said there is no medicine, and mostly the medicine will be expired and we have to take the expired ones. I have to take the expired medicines since there are no new once. so am pleading to them for the children seek they should do well to provide their medicines. And should not wait for the medicines to expire before they bring new ones.

**Caregiver** 2: Tch o3 B/02

**Can you tell me about yourself?**

Am a seamstress and Where I stay is only me and Maame Esi the other child is with my aunty, so we are two, I live in a nuclear family and do not have a good relationship them. Sometimes I feel like just staying alone keeps myself away from them. Yes, where am from most of them are Muslims, both of my parents are Muslims but am a Christian, their behavior and a church goer is not the same. That is why. Yes, me am the breadwinner, head of the family as I don’t get help from the father. No please the father is no more, am left alone. When we are sick we go to the hospital but most at times we go to prayer champs.

**Can you tell me about x? Talk about the girl, how you got to know about her condition**

I was once staying with the father, and the father was a worker, he goes to place to place to work, so we were staying together and he told me he was going to Accra. Then when he went he came and visited us December but when he went back his condition was not good. And his parents called me to come through that when I went I stayed there to take care of him. He told me that we should go home, not knowing he had this condition but he never told me and me too I didn’t know. I stayed there for two months at Swedru the parents place. So I came back and later they told me he is not well. I have to go again and take care of him when I went they were hiding everything away from me I just took patience and I was watching them. The last time they went to want to transfer him then they said we should go to hospital and I should go along with them. That day we went together to Swedru Hospital so they told them if they have informed me about his condition, they said no so they called us together and they told me this is what is happening to my husband so I should also go and test and test the small girl by then I have delivered her and I don’t know what is going on, by then she was two and half years. Small she will fall sick it will go and come, it will go and come, and then the serious one happened to he himself. And I went to the hospital with his junior sister it was then that they ask me to go and test may be I may also be a carrier. I just took a little time and went and tested and I took the small girl too and they said we all carriers so since then they said they will put me and her on the treatment. By then it was too late for him, my partner he didn’t really like going to hospital, he always go for drug store and get treatment and come and take thus all he did often.While at Accra he was seen at Achimota Hospital and subsequently transferred to Swedru Hospital so it was here he was tested positive while on admission. By then the small girl was falling sick often with sore all over the legs and head, sometimes boils. But my small boy the elder is negative.

**How did u receive the news?**

I was looking at everything as if I was dreaming I nearly killed myself it was a pastor and then the wife they talked to me and then the nurse who took care of us was also advising m. she told me that it is not anything plenty people are having it not only me. Through that when I come I go to church the preaching, the advice they give me that calm me down. The **Counseling** It wasn’t ok, and I was not ok. That day when they transferred me to Swedru I followed him with the younger brother so it was there they told the brother and then the sister, that they should call me and tell this is what is going on. They too I don’t know whether they were afraid or what I don’t know, I was sitting outside and they took him inside he wasn’t able to walk he has to be supported. The woman went blank and said it straight to me, he asked my partner if he had told me and he was sitting there. Then the nurse said I told your husband this is wrong with him, he should tell u did he and I said no. that this is happening to your partner. So she said if we do the test we will all be ok. I was not actually counseled I was just informed when she called me in and ask to do the test and it was after the test they took us somewhere that they were counseling us

When I was pregnant I went to a clinic I started it at Mpaha. I go to hospital but they have never said anything they haven’t told me go and do this go and do that. So by then I wasn’t falling sick. It was there I went and delivered that place too they didn’t tell me anything that this is what is wrong with me and the child so it was just one day they ask me to go and test because every pregnant woman is testing so I called him and told him by then he went to work side, I told him this was what I was told but he told that their lying I should not test what am I going to do with that test and I asked him they said when am coming I should come along with you. And he said that no I should not go. By then it was left with some few days for me to deliver and whenever I go to hospital they are not telling me anything and I use to go for ANC, the day I was to even deliver it was the same hospital they did not tell me anything. For the elder boy is 12 years and he is negative so I started the treatment at this hospital and I travel all the way from Mpaha to here for the medicines.

**Can you tell me about how you seek care and support?**

Only the drugs we take from the hospital we do often take and sometimes prayer champs

There is no support from anywhere, It was just a few months ago ehhii the this girls aunty, I asked them so since their passed on am not hearing anything from them they took their not calling me why or they don’t want the small girl, so since then is wasn’t long the aunty. I called her, I went and look for her number and called her so since then she has been sending her 50 cedi’s since she is attending school she will use that to maybe she may need something to buy. But is only once she gave that

So No they don’t come (when am are on admission to visit), they live at Accra and we live at Mpaha. Is only I who call, they have no time to call (to even call), is only when the child continuously asks of the father I will go and look for credit and call them for her to talk to them because I don’t want her to feel bad.

I don’t have friends actually. I dint know why, since my child will be fine I don’t have problem but I play with everybody (I don’t have friends who support me) and for neighbors No please, in our place who will support me there is no one (amidst laughing). Is only my parents who do visit when am on admission?

He (Pastor) does not support but I told him what is wrong with us. He has never even supported me, but he prays for me especially when we are having congregational prayer. He does not come to me house or when am on admission. The support I get Is very poor, I don’t have anything to say is only God that is looking at. I don’t **expect** much since I have not told some of these people is only my pastor I have informed. It came to sometime after the death of my husband I was thinking I will get married to someone but maybe the support to get to care for the child but when I try I never succeed. So when I told my pastor this is what is happening he told me that I should sit alone. Sitting alone is not anything so I should remove my mind from there and look at how I will take care of the small child. And since then I have been staying like that

**What are the roles and activities they take in the care of x?**

When day breaks, I prepare her and send her to school, the school is about one and a half mile away. So I take her and send her there. Then I will come back and if I have small work to do I will come and sit and sow and go back and pick her if time is up and come back and continue my work. In the evening I will stop and when day breaks the same thing. I take my medicine 8 pm even if I will go to church by that time I have to take it, and the child.

**Emotional, spiritual and physical support/coping/relax**

I feel very bad very bad and I pray to god and for god to give her long life, for her to survive, if even one of us life should be taken I prefer to go because the child is young as for me I have reached my years my age I have no use again is only the child am praying for. Am looking at how she will grow and she too will grow and experience life that is my concern. When I feel very bad sometimes and when am disturbed and can’t pray I go to sit and watch TV may be one hour and sleep latter.

Sometimes she will ask me certain questions and I feel very bad, when am crying and I see her I will wipe away my tears and will site as if there is nothing happening just this morning she asked me mommy so the drugs we are taking so all these people sitting are also going to take the same thing? and when are we going to stop taking I looked at her and I told her god will make a way I don’t know what to tell her and I can’t tell a lie too. She knows she is taking drugs sometimes she will refuse she will not take the medicine I have to force her. Because they told us we should not miss

**Disclosure**

(Those who know our status are) Husbands family, pastor, only my aunty I told she is a midwife so when it all happened it was her I called she told me that we should come back to North then I will come and be taking the drugs.

My pastor I have to tell him because when he does not see me he will ask today I did not see you, like yesterday we came from that place to here we did not go to church and when he see you he will ask this lady didn’t come to church why. So I have to let him know. My aunty took care of me since childhood so many things that disturb me she is the one I tell.

(I don’t want to tell) Nobody because telling someone the fellow will look down on you or will not even come near you. Even my own mother who has given birth to me I haven’t told her anything she only know that we travel and come to town. Am not that free with her like my aunty, is my aunty that I always tell my problems to my mother she is not educated if even I tell her she will move herself away from me

**Tell me about the care and expectation from the hospital**

They do well and take care of me. When I come and their not many people they will not even let me sit they care for me fast so I can go. They are very nice to me their ok.

I have never thought (any expectations) of that but for me mine is to take my drugs and go back because I can’t ask them they should give me money to take care of the small child. I can’t tell them anything. Am not well may be I will tell them, it came to a time boils were disturbing us when we came and I complained to them they will tell me when she is sick I should send her to hospital, and we should not sit at home and say we are doing local treatment. We are using health insurance so we pay little.

(I don’t belong to any organization that you meet to discuss) No please

Where am staying if someone is even positive the fellow will not even let u know, because I travel from far to come here for the medicine. If someone know you are having they will spread, you.

(Have you heard of model of hope) No please. I wish I had that opportunity to meet those also living with the condition to discuss it will help me go about this problem.

**Can you tell me about home and hospital medications for x? Perceived barriers to enrollment and side effects of ART.**

I will say is lack of education most of them are not educated and they don’t know the results of how it will end if they’re not taking the medicines. Hmmm another problem is money to come here is a big challenge because for me any little that I get I make sure that the month they have given us if even it is coins I will save it I now have money box then I will be dropping small.

The distance is sometimes a problem, if a colleague hear that u have this you will be pushed away, they will not even like to come near u. so that make most of us to come far this way to take the medications here in tamale.

Skin rashes, she complains about the stomach, if she wakes up you will see her sitting quite, then I will ask her what is the problem, then she will tell me Mama my stomach I have to give her alludrox. Sometimes she will tell me the body is paining her, she is feeling weak.

**What is the most challenging issue/ day in caring for x?**

For me when my husband passed on I tried getting a partner but three men all rejected me just because of this situation so since then if even a man comes I just tell the man to go because the pastor told me why will you keep on telling people to know that this is what is wrong with you. Why not just give up everything and then concentrate on your church and then the small girl?

I always tell my aunty when I meet a man and she will tell me that is good I let the person know this is what is happening to me if the person will continue with me is good if better I tell the person when I go and try it will just cut off

**What kind of discriminations do you encounter due to x condition?**

No one knows what is happening to us and she is healthy now and when she is with the colleagues you cannot know so no one has ever discriminated against us

All the time my husband sister’s husband who is a pastor does advise me how to take care of the child. My aunty is supportive since we came is at her place we are staying she does not stigmatize us

**What are your plans for x in the future**?

Maame …….. I wish to take care of her so that she will to a higher level than me myself, so that she will get to know how to take care of herself.

**What else do you want to tell me?**

I want to ask can’t we get any medicine for the small children for me where I have reach am don’t know how it will end for me my only worry is the small girl. When am no more who can take care of her that is my worry and big problem thinking always I wish I can get medicine which can cure her. And she will be free. And leaving me I will have been happy and struggle till my time comes. That is the only big problem I have. When she grows by all means she will ask me so many questions. And I can hide anything away from her.

And the finance problem that we face, even better food to eat we can’t get sometimes when u eat a day, this morning we only took tea and that is it. I wish we could get enough money so that I can also use it for something to take care of her school, and her personal needs and medicine. That is all I ask.

And instead of her being in school she is here with me, meaning this week she will not go t school making her schooling backwards, and because am here I can’t leave her with somebody. hmmm I have to bring her along when people ask where are u sending this big girl to I don’t know what to say.

Thank you very much

**Caregiver** 3: Tch 03

**Can u tell me about yourself**

Ookk am ………………., am an Insurance Sales Manager, I work with star life, 41 years of ag and I belong to the night of St. Johns International at the church as well as Education Ridge Keep Fit Club. We were born 6 in number and am the fourth born of this family thus, 3 boys 3girls so far we lost one boy and then a girl, so we are now left with four. The same number 2. 2

My Extended family is in Navrongo, and in the nuclear family here (Tamale) I do but the rest are out of so unless is extreme then they discuss it and then we will see what we can do

We are not so close together (with the entire family) and not so well but is normal. I go straight to the hospital because as a sales person I have to get quickly and know what is wrong with you, you treat it and you move

**Can you tell me about x? Talk about the boy, how you got to know about his condition**

This is about the third year since the parents passed on. (He use to have) sores that will not go for a very long time by then ehhii it was only the father who was tested and then I was told it was positive so I encouraged him to ask the wife to go and test, so after she also went and test she didn’t want to come out clear as to whether she was positive or not yeah, but ehhmmm my little background I was teaching science , I realize that the sores that were developing on the small boy are just too abnormal, around the ears, sometimes the mouth, most of the sores had bad water, very very smelly, so I asked ehhiii the father to test it but it was not forth coming. They concealed it from the whole family. whether he was positive or not, we didn’t know. soooo, By then I have to begin questioning certain things on my own, whether when the mother was pregnant does she should go for clinical and I got realize that it was no. Then she gave birth I was not here I was then in school UDS, so I made a follow up to find out when she went to give birth wat happened and then I learn the nurses were very hard on her, because she was not coming for clinical (ANC), so I presumed that she was hiding something from the family, then when she herself finally started going for the retroviral drugs it was affecting her because she was not so religious taking the drugs, so I started suspecting but I have no proof because she will not allow me to get closer and go and test the boy, so my only proof. And then where I had complete access to the boy was when she died April, 2017. And had ehhhh total care for the child after the burial the first thing my mind was is to be sure of what is really wrong with the small boy because the sores on the body was just too much. And that was, because of that I took the responsibility to bath him myself because nobody can touch him because the sores was too much that when you are even bathing him the whole neighborhood will hear so after two weeks I carried him to the Central Hospital and they asked me to go for blood test and I went for the blood test and eeehhh madam Abibba and the other woman, eehhh that colored woman I can’t remember her her name she also one of the workers there. Told me that oooo that I did well by bringing the child, he is positive. Was she on any medication and I said no if he was I wouldn’t bring him because the mother never disclosed it to us. So from there the medications started. So I went for the first, second month so I said no I have to let somebody to know about it.

Yess ehhmmm they, they congratulated me and made me to feel is ok the way they they they welcomed me and then they told me that I have done well they have never seen such a respond. especially I being the uncle and not the real father. That I should not feel worry because whatever they are going to tell me is controllable is not out of control. So the counselling was good which help emotionally. The counselling ehhiiii? They told me we needed to do it in phases and I made them to know that eeehhh I have already seen it before because my late brother was having it and I use to go to Teaching Hospital to take the drugs sometimes send it to him at Accra so if there is something like that they should not hide it away from me. Because I have seen it before. so they say if that is the case then that is what was happening.

**Can you tell me about how you seek care and support?**

Actually I ask the Counselling Unit some of these questions that if he is sick what do we do and they told me that. If he is sick he will go through the normal treatment as every human being. if is malaria we treat malaria but we should avoid any herbal medicines as much as possible. So I ask them of all those pertinent issues and then they told me that but I just made it a point that the way I seek medical care if am down with any symptoms am not sure, he will also go through the test and they will treat him. (I don’t really get support) what strengthens me is the way the boy has recovered he was, it was just very very bad, so at the beginning when he started to take the retroviral. It was not so steady like I expected, but after 6 months on then I drive some motivation. I think the support from the family is the ehhh, all of them know that when I came I made to know that, we call him Akor in the house he will take his medications at 8 am then the next morning any time before, especially before when their to go to school so the mother makes sure by 7:30am he has taken his breakfast. (yes I get support) from family yes especially physical not financial support, but support from friends and neighbor’s no. The support am happy because ehhiii he never lacks the drugs because he has a senior most brother and my brother too here, so I made them to keep what do u call it the record, so that when am in seminar and the next date is tomorrow, tomorrow next when am not there they can try and get it so that he does not skip, I just don’t want him to skip

When am not ok, I make noise and let them know that I just don’t want the guy to skip.

**What are the roles and activities they take in the care of x?**

Yeah you saw him here he is more or less like my friend so I make sure that I let him know that look you have to take your medication by 7:30 am. So by 7 am … when I wake up by 4am and am getting prepared for work by 5 am if is school days by 5 am their up. He sleeps with the other brothers. That’s their room (pointing there). By 5am if I wake up and am not seeing him, I have find out whether if he is well, if he is not well then I will detain him then we take him to the hospital, make sure that he is but when he, his well he has to take his bath take his breakfast, dress and I will show him where his medicine is on this first drawer. he takes it. They will leave the house before I go (amidst laughter). They will have to leave the house before I go. Yess

**Emotional, spiritual and physical support/coping/relax**

You know it came to a time ehhh before this thing, sometimes when he goes to play with his colleagues outside the sores were so much that he will be walking and this pus, so he will come and tell me that uncle Emma the children say I should not play with them because I small. Yeah so once in a while I so I tell him Akor you see this medication you have to take it seriously if not this your body it will start smelling and your friends will insult you and you will come and complain will like to to be like that and he will say no so he himself it pricks him. So when is even pass 8 am and am not there or the mother or the brothers the the the has brothers who have gone to their ground mother, they will remind him that have you taken your medicine Yes, we are Catholics we say our prayer in the morning and we agree that everything God is in control. Physically I cope yes that is why I belong to a keep fit club

**Disclosure**

Naaa apart from the family members no, I don’t have anybody knowing about the condition. Wish to tell ehhmmm is it the family or the nuclear family. Eehhhh I think if I will like to share is may be if I have friends who are medically oriented and I will tell/seek them tell them to see whether there will be a better health care for the boy but ehhh the way society is, even my extended family in the village I will not like to discuss with them because they will even think that the rest of the family are HIV

But I really don’t want to tell others, No I doubt I doubt. Ehhmm I think my friends I don’t want to tell them at all. Yes, I don’t want to tell them. I don’t want to tell them because, I wouldn’t know what they will tell the boy and I don’t know how he will handle it because he does not know why he takes and he may tell his friends. Don’t you know am like this and then before you realize it has spread and he is ehh the friends may like to be with him but the parents can say no

**Tell me about the care and expectation from the hospital**

As for Central Hospital I think if am grading it it should be the best in Tamale as compare to when I use to go to Teaching Hospital. Teaching Hospital is just chaos they don’t have time for the patients and don’t even know what it is about. Yes, this unit. Nooooo noooo because of that I have adapted Central Hospital for them. ahhhaa and the handling (there is good). And because of the advice they gave me at the counselling unit I have taken extreme care so that he doesn’t even fall sick frequently. I can’t even remember the last time he was sick. my brother is here.

My expectations Yeah I think what we need to do is to decouple those even if yeehhh there will be a unit to screen to find out whether somebody is HIV, if it is that then there should be let say place where the blood sample test will be express, if not for instance when I last went there we went in the morning if not because I took leave and as a sales person we have 10 days leave in a year .so have to take three days. I f not if I wanted to go work I can’t go to wait up 4 hours to take. Ehhhh is frustrating.so with that if the fellow think of going to sit down for this results yeah is good. health is good but you are also looking at what the other family are depending on you so if there a place where the suspected cases you go; they simply take the sample the next one-hour you get your results. Then you go to the unit for may be the counselling process and subsequently the medication it will be find but if you have to wait joined the normal que, for everybody to either, it’s too frustrating

Nooo belong to any NGO, institution that is concern with PLWH

**Can you tell me about home and hospital medications for x? perceived barriers to ART and side effects.**

Aaahhhh you know from my experience with particular reference to our problem, the father was positive the mother too was you see and we have realized that they need constant counselling, for it came to a time their behavior looks like as if they are not normal let me say they easily become angry, something you didn’t mean what you were they feel how, they easily get time to think the the disease as how me in this state so like my late brother’s wife like this she just gave up completely. (means because they were not counsel well or because they did not accept the condition that is the reason why they didn’t put the child forward for treatment). I think ahhhhh you see it,,,, they were kept the child and were not counselled. As for my brother he was open to me ok the counselling they gave him it was ok but you know he is a man but he was having another wife in Accra too so he will take the ART he even has a baby with that woman after he was taking the treatment but because he was regular that child too was tested and he was negative and the second one is negative up to date. Ehhhmm laughing that one is general because I think like I told you is like the problem is in the testing stage if they think they are frustrating and wasting their time, they easily become angry and they go. So they look at the time they will go and waist there and the people will speak to this to them, they don’t like, l don’t have time. So once upon a time like I even ask the boy before I gave him the first medication whether he has ever seen this drug before and he told him yes, I said where that the late mother use to remove her own and breaks it and give it to him. So like they even start self-medicating the child. So sometimes they may run-short of the quantity they have given them for the duration, and when they go depending on the counselling center, they are if they are they don’t give them. They come and hang. Like the mother started with TTH, before we realized she moved to Savelugu to take. Ehhaaa So is like Savelugu was more accessible and more private than aehhenn

yes it will very private, the medical activities should be express (faster) ehhhh they don’t like where so many faces see them yes yaeh like sometimes when I go to the counselling unit to take the drugs if not because am determined and you sit at the waiting site some of them they don’t feel comfortable looking you see others turning ehhaaa they feel like as they come paah enters take whatever they need and depart. So like the doors can be two, ok you come you enter when you finish you passes this way. away (the structure itself can be a determinant factor). I think if there is some kind of inducement it will pull a lot out of their hideout to come for treatment.

**medications side effects?**

I think when we started eehhh it was latter when I asked him he told me that the mother use to break her own for him, so the first medication he actually reduced you understand but the sores started healing but he was he reduced in size and always look sick and dull but with time they changed them and the dosage. Then we took his weight then I as you can see him now he is ok, those days even if ehhhmm he was to take his bath ehhmmm the corner of the ears you could see the sore but now those things have gone.

**What is the most challenging issue/ day in caring for x?**

Yeah yeah I aaaa you see the I keep on telling you when very one year two years if you see the sores on that boy eehh the whole house even they were even medical people they can hold him but I have the courage to to to dress the wound , because I have the mind I bought my own gloves, I bath him and the moment I can’t always forget is when he cries and mention my name and then I told him that it will be ok

**What kind of discriminations do you encounter due to x condition?**

Ooohhh no no no

**What are your plans for x in the future**?

Ehhmmm the problem I use to face is when his sores were on and he will come and report to me that that his friends will say he smells. but now he is ok but the plans I have for him is the society in which we are in Ghana eehhh in the near future I don’t know if the public sector will stop to discriminate against people living with HIV. So am trying my best to get identify his talent, what kind of job he can do with his own hands so that when he is well educated in that aspect, even in my absence he can be on his own let say that is my plan (He should not be a dependent). Yes, he should not be a dependent because as soon as he becomes a dependent he will not take the drugs because some of them from experience even they feel they don’t have lorry fare to come and collect the drugs and you know when they take they eat a lot so I don’t joke with his food laughing. so that is my worry now I really want to educate him to a level where even without government work he can have his own job

**What else do you want to tell me?**

your study is a welcomed one, because honestly speaking am from Upper East and from my experience you go, see them and why am adding that aspect is that there were others I think those days they belong to one of the NGO, they give them food to take but when they started wining them away from the food they also stay away so you see. So this will have informed eeehh official, stakeholders that there should be an inducement to bring those who are not having. Some of us are courageous and want to see that tomorrow…(So when u say inducement financial/physical) I don’t know financial, fine but you, need you see because of them when they take the drugs they eat a lot so u can give him money to take, let say ten cedi to take lorry from where he is coming. But when he /she go home and he is not able eat because the drugs he is taking he/she may reduce the quantity he takes or will not like to recommend it to somebody, because you can’t cope with the feeding. So is better they stay away

That bring an end to our conservation, am most grateful for your support

**Caregiver** 4: TTH 01, 29/10/19

**Can you tell me about yourself?**

My name is ……., a business woman, selling provisions and I have four children and myself five. My first daughter is the head of the family and she is a nurse and now she goes to school. We live in an extended family but I now live at a rented place at Vitting due to family quarrels and argument. And I don’t want quarrels; I want peace so that I can care for my children. The children father is not there (passed on) so when I live in the family house everyday there are quarrels. That’s why I went and rented a room.

The second daughter also a teacher helps the elder one for decision making. We go to hospital sometimes and other times we go to prayer camps. is far I travel to different towns such as Kumasi for prayers, I have send this child two different occasions for prayer at prayer camps.

**Can you tell me about x? Talk about the girl, how you got to know about her condition**

since birth I have been caring for the child so for almost 12 years and since she was diagnosed 6 years ago I have been the one caring for her since the father died of the condition**.** Six month aaahhh six years ago that I got to realized she is sick. she was eating food one afternoon and the face closed and she collapsed and fell off, then we runned and brought her to the hospital (TTH). When we brought her they took care of her and told us she did not have blood. So they are going to give her blood and she was unconscious when you prick her she does not feel anything and was not talking. She laid like this for almost two months at the hospital. They gave the blood and even kept her on oxygen. First we sent her to SDA**.** Then from there they transferred us to TTH here**.** She got better and we were discharged home, then it came to my mind that since the father had passed on by this disease and I also have the disease it will be good to test her too since she was falling sick often

The father was sick and we did not know what was wrong with him, then they did this test for him, then they confirmed that disease was on her. Then he came to die of the disease**.** So after his death how many years or month did you get to know this child is positive? Sick years after his death that this last born started falling sick eehhhhh while the father was alive the child was even falling sick often though. I brought her for the test on my own after being discharge Because of the way the father died and falling sick often likes the child. So let me come and check may be that may be the cause

When we went to the lab and they did it they gave it to me to bring it to the nurses here

And they confirmed the disease was on her (the other child too I want to check and be sure even). The way they said it(status) was comforting but I was crying and she consoled me in the morning. Hmmm what can I do for the crying there always I cry

**Can you tell me about how you seek care and support?**

When he is sick unless I talk to the elder daughter because there is no one who can help me. She is the one who will give me money. Send her to hospital, this one aahhh (TTH) the elder sister wants us to come here. When she is sick for instance malaria we go through normal procedure, here is just the medications. They don’t (behave badly to us) since they don’t know her status, is only here that they know. So here they told us that if she has malaria or anything we should go there.

So when we go there they attend to us normally. Gives us medicine and those not there is written for us to buy. only your daughter that helps u, in which way does she help you, the second born the teacher also helps

They(friends) don’t know of our status. But when the child is sick and am at the hospital they come to greet me, they sometimes bring food but no money and as well they pray for me and consoles me. They (immediate family) don’t help, (What of assembly man and Imam) No for the imman he come to pray for me, and sometimes prepare water pray over it for us or sometimes some concoctions as medicine in Islam for us to be drinking, for this he has done well paahh

The help I get from this people, Is it ok. It’s not enough if I get other support it will help me. Rent for instance for my family, money for business to care for them help in the form of food supplements

**What are the roles and activities they take in the care of x?**

I pray at 2pm, wake up at 5am and we all pray, then I cook for them to eat and go school but for this child I help her dress up and prepare, make sure they go to school, go to shop, at 2 pm I may go to market to buy food items even though I often buy much at a time and often when I want to close. She takes her medications after food.

Because she is always sick I have to help her do things fast to go to school? As such when I see she does not want to go to school I will know she is not feeling well. I need to console her, give her medicine, and then do everything so that she goes to school

No I don’t have a friend who will bring me problems and belong to any organization that is concerned with HIV.

**Emotional, spiritual and physical support/coping/relax**

I drink medicine, sleep and pray and I and my children watch TV. No (I don’t chart with anybody)

**Disclosure**

Is only the elder a lone I did, and not tell the rest? Because the elder one is matured and takes care of us. And those not want what is going on the younger one know and it will have disturbed them. She said the children are small and if they know of this it will disturb their minds and since their going to school it will affect their performance. Nobody knows

I don’t want to tell anybody curaahh because of how the family is I will not tell. You see that we have to park out of the family they will disturb us paah if we are there where I want the children to reach in future they will not reach

**Tell me about the care and expectation from the hospital**

We collect medicine only; first they use to give us food stuff long ago but they have stopped they say the food stuff is not there again. It was helping us paahh. Yes, good, here they do well (and takes good care of us). When u come they receive you and they don’t shout on u, they only advise you not to miss the that, but when the date is passed and you come they will speak to you that you should not do that again. They take care of her and write others to buy or collect by NHIS (when she is normally sick). They don’t know her status so no bad experiences has been seen. No (I don’t belong to any organization)

They should help us with money for business to help with the care or food stuffs, help in respect to their schooling and materials for learning like books. So we need help paa to help us small for the children wellbeing. Rate them excellent they are good paah, they take care of us very well.

**Can you tell me about home and hospital medications for x? Perceived barriers to enrollment and side effects of ART.**

Some is money for transport may be on the day their supposed to come for the treatment, some too are afraid to come and see others here (stigma), others too do not now the important of the medicine. Also disrupted medicine supply makes them go and not come back again. so some may want to travel so may want two months but due to low stocks may be given one month and go and travel and cannot come for treatment. So they should help for the children medicine to always be there enough.

Vomiting and eating plenty. At first she uses to vomit whenever she takes it and nearly prevented her from taking it, they reassured us and it stopped alongside but she eats a lot. And no money to buy food stuff

**What is the most challenging issue/ day in caring for x?**

She went to school and one of the younger uncle’s child small girl told her she should be careful and think of herself, HIV killed her father and that they can’t play and eat with her again. It hurt me I wanted to call the child father for us to talk about the issue but my elder daughter said no because is the family member, I should not say anything. My child did not say anything she came to inform me and I told her it’s not true. Then her mind came down.

**What kind of discriminations do you encounter due to x condition?**

Yes, it happened once, that was the day the girl said that she may be positive and that they will not eat with hear again, but when I told her is not true that was all

**What are your plans for x in the future**?

I want her to go to school, finish and be independent care for herself and become big, because now am doing small small to take care of them to go through her education. When this happens she can’t carter for herself and me too in the future

**What else do you want to tell me?**

The help that is what I was saying its monetary problems, we want money to do things and care for the child, food, their school issues. Does what we hope people should help us. They should get us some money to help us care for the children

**Caregiver** 5 TTH 02, 1/11/19

**Can you tell me about yourself?**

My name is Fawaz, she is my junior sister, am the one like who takes care of her, am a student and have business that I do, I rent canopies. We are 26 including me, hmmmm I live with my mother, ground mother and my father is late. Meanwhile my mother is seriously sick with a disease condition called……. And for my grandmother she is too old and I take care of all of them. Am the breadwinner of the family and usually makes the decisions in the family except serious ones that I consult my uncles, we live I a harmonious family and usually have some few quarrels. When someone is sick in the family, because of schooling and work I usually takes the person to the drug store especially my junior sister for medications, if am not seeing any changes or changes I will then and the person to the hospital. No we don’t go to prayer champs but for Imams we go because my mother’s conditions need spiritual prayers and sometimes I do send the girl too.

**Can you tell me about x? Talk about the girl, how you got to know about her condition**

My mother use to take care of her until two years ago when my mother fell sick with that condition and could not take care of herself and my siblings. Ehhiii there is this I don’t how will I say it, like this in Daghani we say arizina en disturb ooo. Ever since this happened I assumed the responsibility of caring for them and is ever not been easy for me. Like she has been falling sick often and sometimes she will complain of her stomach and all that we thought maybe she will get better and because she is an albino that’s is why. But her condition never improved and became so serious about a year and half now. I first I brought here (TTH) and had them ran some test for her especially hepatitis b because of the way she was growing lean I thought that was the cause. After the test turned to be negative so I made them do HIV which to my surprise she was positive. I was wondering if she was raped or something cute her, hmmmmm

(Were u counseled before the test) No please I was not counseled at the lab there. When they gave us the results they said we should come back the next day and we did. And they referred us to this unit and maahh counseled us and she was kept on the treatment. I was shocked but not really shocked because when we came from looking at her the way the body was showing too slim. So I knew something was wrong with her. So after the results were shown to us though I could not really believe it I wasn’t really to shock about it. I, I, what will I say hmmm, I don’t know to put it but like I said before we don’t usually go to the hospital immediately because of my work and schooling so I often send her to the drugstore for treatment before sending her to the hospital if it’s not getting better. Especially when she complains of stomach pains I get her medications from the pharmacy if is not changing I will then send her to the hospital.

**Can you tell me about how you seek care and support?**

Hmmmmm, my father has passed on and am to care for the immediate family is hard for me. I do not get any financial help but some kind of support from my immediate family. The problem is those closer to me are not in good frame to help much for instance my ground mother is too old and frail to help and my mother has this condition that destabilizes her way of life, especially when the condition becomes serious and I have to absent from lectures. I do get help from my younger sibling though. I do get help from my ground mother who encourages me and advise me on certain things. And my uncles who will help with talks but sometimes after harvesting they give us some food stuffs. And they are always praying for me to be able to care for the family since my father is late. Yes, my friends do help, especially my two close friends, they help to emotionally motivate me and talk to me when my mom and sister condition becomes serious and take me away from school. And sometimes my school issues. Yes, they (neighbors)support me but not a serious support. Usually when we are on admission they do come to visit and pray for us and bring food for us. (community leaders) No, He (Spiritual leader) prays for us but when we are on admission he does not come please.

Their support is ok but not really enough because of my situation being a student and having these dependent on me, I wish I could get more support

**What are the roles and activities they take in the care of x?**

Hmmm is serious please, usually especially some days when my mother sickness becomes high its bad very severe for me, such days I have to take her to the spiritual leader thus the Imam to pray for her. So when I wake up to pray, they wake up latter and the other sister will help prepare them for school while I see to it that my mother and ground mother are fine, I then give money for their upkeep which I give them 20 cedi for a week and on weekends I give 2 cedi for a day. The girl will take her multi-vitamin called vitafol and septrin before they all go to school. Then I will prepare and go for lectures hmmm most often am late for lectures. I return from school early to go to the rental place to do business and come back to make sure their feed. I ensure they bath and she takes her medications before we all go to sleep. But I have to be alert for anything because of my mom condition as well as my sister. hmmmm sometimes I have to skip school and bring her to the hospital and for her medications here and my mother and ground mother. For the market to buy food stuff often my other sister goes as well sometimes to make things easier I do go to buy them is difficult but I have to

**Emotional, spiritual and physical support/coping/relax**

Like sometimes I will just be playing with her for not to be feeling like she is sick or marginalized and it helps me too feel free and relax I must tell you. And I don’t want her to feel like am distancing myself from her. Even when am not around because of work and am worried about them I call to talk to her to be sure their fine. I do pray often too and often ask for prayers from my imam

**Disclosure**

My ground mother only is the only one that knows about her sickness, Because I don’t know others will take the news, especially my mother who is not in good frame of mind. If I break that news to them I don’t know how they will react, how they will feel towards the child and they will look at her. Hmmm (I wish I told) my mom, because she is sick, I don’t want to burden her psychologically. There are some people in the family I will not tell because I don’t trust them, and I don’t want them to know. Because they the kind of people who talk a lot. So when they know something like that they might go around telling people this is what is going on with this child

**Tell me about the care and expectation from the hospital**

The care is good, they treat you well, they respect us and every information that you are suppose know they will give you. For me because of my busy schedules I will expect them to come to our houses to give us the medications and educate us

**Can you tell me about home and hospital medications for x? Perceived barriers to enrollment and side effects of ART.**

Some I don’t know but they feel like is waste of time. Like they don’t have time to come for the medications. She complains of the body itching. She does not complain vomits like others do

**What is the most challenging issue/ day in caring for x?**

Sometimes the challenges are like I have to skip school, care for her, and bring her to the hospital and all that. It hard for me especially concentrating on my studies

**What kind of discriminations do you encounter due to x condition?**

Hmmm this is difficult for her now, until she was diagnosed and I told my ground mother she use to eat with the other siblings but I remember one day when I got home. She complained that her ground mother does not want her to eat with her siblings again and request she eats alone. And when I asked the ground mother she said the disease will spread to the siblings. And since then she eats alone as it was difficult to convince the old lady. And the way she looks slim the other children don’t really want to play

**What are your plans for x in the future**?

Hopes she gets well and like further her education

**What else do you want to tell me?**

I just need more support to care for her, like money support, talks, advice and all that.

**Caregiver** 6: TTH03,

**Can you tell me about yourself?**

Am a teacher, and (don’t do any job or business side that) No please

We are six from my mother side and we have other family members, my father had two wives and she also has six children too so we are 12 children, Am the fourth born from my mother

Ehmmm My husband is not working and am the breadwinner, makes decisions and head of the family. No (we don’t quarrel) their please their supportive. I go to hospitals and sometimes I go to prayer champs, there is a pastor called King Smith in tamale here that I do go to often for prayers for myself and the girl when sick, and couple it with going to the hospital, I don’t take herbal medications

**Can you tell me about x? Talk about the boy, how you got to know about his condition**

That is my first born and I have been taking care of him since birth. After I gave birth to him, I was then in Accra he was not getting well like, any time he sucks he will vomit again, when he sucks he will vomit again, so we, where I delivered the doctor gave me a letter to send me to Korle-Bu. So when I went to Korle-Bu their doctor there then they tested, they run a couple of test for him. They told me well, one in charge there counseled me and they did the test and informed its positive so I should also go and test. He was then around two weeks old, so I also went and tested and it was positive. So that was when we got to know so we spent about a month in the hospital, after they discharged her they started his drugs.

(when I was pregnant) I was consulting in a private clinic and I told the doctor I been seeing some on TV, they said the transmission of from mother to child can be treated so he should test me. But he said he has not seen those symptoms from me so he didn’t do that test, I told him that he should do HIV test because I have been seeing on TV that they want to stop the transmission of from mother to child but he told me he has not seen such symptoms of HIV in me so he wouldn’t do the test. So he didn’t do. Me too my mind wasn’t on it, since he is a doctor and he says he has not seen those symptoms me too I took my mind off it and didn’t do that test. Till I gave birth and the child was sick. So it was at Korle-Bu that I did this test. Hmmmm I was scared and I was down. I was devastated ehmmm I thought my whole world has ended then I was afraid that the child will even die, yes so I was very worried but they told me that that wasn’t the end of life. If I take very good care of myself and take care of the child, we will live long.

Ehhmmm he (my husband) wasn’t told it was I they tested they didn’t test him. I couldn’t inform him. But latter they tested him and he was negative

**Can you tell me about how you seek care and support?**

I send him to hospital and sometimes he is admitted and other times he is not. Where I was initially working they use to pay me well I used all the money to pay for the bills by then I was in Accra. So when he was two years I fell seriously sick to the point of death, so the doctors who were seeing me said I need to be hospitalized to be treated so I should go back to where I came from. I should go to my people so that my people can help me with the child and then they will hospitalize me and treat me so the doctor also wrote a letter for me so my sister came and park my things and we moved to tamale here. And when we came I was admitted at the second floor TTH here and I was treated and recovered. Then was discharged.

For the small boy after I was admitted and discharged after a month he was also admitted at TTH here and that was the last time he has been on admission.

Hmmmm my sister sent money; she lives in England so she sent money to my elder sister to take care of me and the child. Aside my sister who supported I had a late sister who had friends from the Netherlands they also happen to visit and they meet my brother and asked of me and he told them am in the hospital and they even said I needed blood. After I was discharged they came home and we spoke, they ask me if it is this I should tell them they will be helping me. So I also told them is like that. They said every month they will contribute and send to me. By then I was sitting in the house I was not working because I have left my job in Accra and I came so they were helping me every month they will send me money through western union. To go and cash for me and my son so that was what we were using for that time till I recovered fully and had a job. They were sending me the money for more than a year and half so the second year I applied for a job and got it so they stopped after I informed them.

I use to have friends but after I went to Accra for sometimes after I came back they all left tamale, they were my childhood friends since then I have not had any friends. (neighbors and community leaders) Noo ooo, while on admission is only my sister that came no one else

I don’t have anybody to help I live in my apartment a lone and nobody helps me. For (my pastor), I go for prayers but he does not know of my disease and the child, unless I tell them in the church so when am sick and they cal I simply tell them I will come I don’t disclose its them

(look at the care and support you get from the family) Initially they use to…. How do I say this when I was on admission and left my son, because of his vomiting problem they use to not to take good care of him my senior sister like when he is to vomit, imagine you all site down and you are eating and then someone suddenly vomit you will fell disgusted so they use to be insulting him and that time he was two years. Hmmm they use to insult him and they don’t want him near them sometimes they will be sacking him where they are but a brother was also there. I didn’t know, he came and told me that this is how they are treating your boy. And did stay in the hospital for about three weeks so when I was discharged everything was ok. They didn’t tell me this was what they were doing or that. Or my son does dirty things. And me too I didn’t ask them so we were all together. The support is ok but how they treated my son was bad in my absence

**What are the roles and activities they take in the care of x?**

Ehmm sometimes its hectic like u want him to, like his problem is eating he doesn’t like food, as for bathing and other things he will do it but you have to tell him several times. U will tell him take your tea and he will be sitting down take your tea and he will be sitting down. When you tell him go and bath he will be moving slowly even his teeth he will not bruise unless everything you have to tell him, everything do this, wear your clothes ehmmm then tell him to do almost everything while the in the morning what do u do

Am always late to work because of him because for the junior sister who is ok (negative) she does things fast fast fast she you will not even tell her, she will prepare herself and be waiting for us. For him you have to tell her everything, so I always talk, talk shouting talking shouting if he is done then we will leave. We always late and he himself always late and he himself JHS 1 they give numbers at 6; 30 am he leaves the house at 7: 30 am and gets to school around 8 am, so the last time he came and informed him that they caned him for late-coming so I should try and talk to the head. So I also went and told the head that the boy is is not well and he can’t come alone I have to bring him. Where we leave from there to the school is far and before I will finish and bring him time will be gone. So they should bear with and leave the canning. The head said he has heard and that some teachers are very strict when their very strict when they are on duty whatever you tell them they don’t listen but he will see what he can do. So these days he tells me to wake him up early. So I wake him up around five, then he takes his bath, takes his tea and prepare and leave. I look for car for them and they leave before I go to work. He takes is medications in the night because the drugs we take is only once in a day and when we take it we feel like drowsy so it’s good to take it bedtime when we are to sleep.

And when I close I have to came to the junction early because from there to the house is very far so I use to let him come by himself but since he is suffering because his books are very heavy they are many so these days I go to pick them because I close at 2 pm so I tell him to wait for me so that when I come I will go and pick him and bring him home. Then when is 3pm I will go for the girl

**Emotional, spiritual and physical support/coping/relax**

I just manage, am suffering, I sometimes prays that god should give us strength; he should let him recover like. One day we should just wake up and hear that there has been a cure to for the disease so that we will be treated and be found. Sometimes am scared because I don’t know what I will tell him when he gets to the age of knowing the status. Sometimes I want to tell him but I don’t know how am even going to say it to him that you are like this, you are like this. So I always pray that that day will never come. (Physically) I really like wrestling that what I watch on the TV to take my mind off it. Here I don’t have anybody because am too busy, look at the time am coming (the respondent had arrived at the time the unit has been closed at about 2:15 pm). So I don’t really meet anybody here who may have my problem that we could share our sorrows. But way back in Accra we use to have groups and we meet and talk and sometimes we go for meetings and then nurses will come and educated us on the condition and other things but since we moved to tamale 2008 up to now I have not had anybody to discuss this with the person. Those meetings were very helpful and I wish they are done here too.

**Disclosure**

My sisters and their children are those who know about our status didn’t tell their children about my status but my sisters told them their children are older 26, 30 years. They tell them your aunty is sick of this, your aunty is suffering from this. Hmmm am ok with they (sisters) disclosing (amidst laughter) because they come to me, they visit me, I don’t live with them but they do visit me. Sometimes they stay for about days with me, we cook together, we eat together they don’t do anything, ehhh stigmatize me do anything to me and they are close to me, they do everything with me they don’t do something bad to me and the boy. Ehhhiii my sister, my sisters I told them because the point that I reached I thought I was going to die and I don’t want to die without telling them the truth because if I don’t tell them and later they get to know my son may be deserted so I have to tell them that this is what is wrong with me and my son

I didn’t tell my husband because we were not married and we were moving together as a couple and that was why I didn’t want to tell him. I don’t know how he ill feel then also may be if I tell him he will run away and leave us and will not come to us again and the boy will be asking for the father and I will not know what to say and more to the point we were not married and nothing was tying us together ehnnn. So if I tell him he can easily leave.

He(husband) know now, ehmmm one time the boy went to spend holidays with him in Accra and he said nooooo he doesn’t understand why this can’t he eat and he is not growing like a normal child and if you look at his age and his body he is not growing well. He said he will send the boy to the hospital, so I told him not to go he should wait. And he said noooo he will go so he sends the boy. So I said he should send him to Korle-bu. He already has a file there so he went and fortunately he meets those who use to take care of us, so they told him everything and then they tested him and I thought he will run away from us but he still come to us. Am not with him again and we are no more together. I don’t have anybody to tell, all those I wanted to inform are now aware. No one, you do not want to tell, No one

Those who are not part of our immediate family I will not want to tell them, those outside our house you know in the villages we have various houses in the area so our dierr they know outside the compound no and I don’t want them to even know.

**Tell me about the care and expectation from the hospital**

Anytime I come here they take good care of me well. And sometimes when I come and people are there and am in hurry I tell them I asked for permission and I came or I used my break time, sometimes I don’t even ask for permission, and the break is 30 minutes and I use the break time ride here and when I tell time I beg do it fast for me they help and attend to me, and they see me and I leave. I would have wish they give multivite or ion or drugs that will help patient to eat well or keep us healthy. We buy such drugs, as I just came I told the guy I don’t have money to buy, I always buy two parks, containers but today I don’t have money. Where we take the medication inside there. so today I told him I don’t have money, they have not paid me yet and he said I should take it and send the money to him latter if am paid. Two parks one for me and the other for my boy. And I see that when he, my son takes it he eats well. So they could have been sharing for us free. When I was in Accra they use to give us tom brown and other things but here they have never given us these but one time I lent they were giving corn and other things but I never had some

**Can you tell me about home and hospital medications for x? Perceived barriers**

Because they don’t want people to know, because of the stigma they don’t want to come and meet known faces and then after that they will be treated like outcast thus why. And some too go to other regions. I know somebody from here who goes to Bolga for the medication and as well know people from Bolga who travel here for the treatment. So u may see people who are sick of the disease and don’t come here but that does not mean they don’t take the medicine. They go to other places for it. Some of them are also seriously sick and ca not come for the treatment and do not have people who will come for it. Others too don’t have money to take taxi for fare. I use to meet people here who complain that they walked here and they don’t have the support to come here. And I use to give them money for transport because I was getting support from the Netherlands people so I supported them. And others are weak and can’t walk here and can’t move even move around

Vomiting, feel drowsy and sometimes he will tell me his eyes are turning, constant headaches that he most often complains. Always he complains of headache so I will tell him that you don’t sleep enough that is why so sometimes he will sleep as early as 8 pm up to 5am and wake up. And during weekend he may sleep up to 10 am and still complain of headache. And tell me that that these days I sleep early but my head is still paining me. Even yesterday when he came from school he was complaining that his head is aching him. These are some of the sick effects.

**What is the most challenging issue/ day in caring for x?**

Ehmmm yess, because of the way he is when you see him no one will tell you he is sick but they don’t know the sickness sometimes when he goes to school they give him names they call hin “chingilingy” eeehh “yiateyiate boy” what and what ??? people just say all sort of things, sometimes he will say he doesn’t want to go to school again, sometimes when he sleeps and wake you ask him to take water he will tell you he doesn’t want take “I want to die “ yess I tell him when u get up drink water so that u will be fine. And he will say leave me I want to fall sick, I want to die I don’t want to take I will not take the water, leave me alone. Sometimes these things worry me. And I will have to leave him to be then when he sees that I don’t give him attention then he will start doing what I tell him to do and we will be friends again. Sometimes u will see that he just be calm. Sometimes too you will see that he will tell me mama when this medicine finish don’t go for some again, I don’t want to take I want to stop why do you give me medicine all the time. When will I stop taking this medicine? When such questions are asked it hurts me because I don’t know what to tell him and I feel sad and I also pity him because is my fault, hmmm I didn’t also know. So when it happens like that I just move somewhere and cry. I pray to god that he should make a way for my son.

**What kind of discriminations do you encounter due to x condition?**

Aside being called names at school and the issues with the aunty despising him, he plays with the junior sister in the house. We live in a self-contain house so he does not go outside to play. They don’t even go out to play with other children but sometimes friends will bring their children and they play together, that one no discrimination. Yes, she is the second born but she is fine an is negative, and he is first and positive.

**What are your plans for x in the future**?

Hmmmm his future I want him to be to become a successful person and then his life should be a living testimony for other people

**What else do you want to tell me?**

Hmmm like I was saying the multivitamin I wish it given free as well as drugs that will help them eat well. If they can provide us with that, that will help the children to live well.

**Caregiver** 7: TTH 04

**Can you tell me about yourself?**

am a typist at Fuo, and don’t do any work aside that, no please, I and my husband we rent so we don’t live at the family house. We three, my child, husband and myself. He(husband) is a tailor, Yes, we relate with them (the extended family) well they are at Daghandafuo.

My husband is the head of the family and the breadwinner is the one who makes decisions. We don’t have good relationship with them, the extended family. We quarreled because of the sickness. We go to the hospital and don’t entertain going to drug stores as we were told straight the unit here.

**Can you tell me about x? Talk about the girl, how you got to know about her condition**

She was sick and we brought her to the hospital and they let us do the test and they told us she was positive. And they said I should also go for the test and they said is from the breast that she sucked, so when I also went and test they said is positive. When we came here (TTH), we were admitted for about three weeks and they ask us to go and do the test, because of the girl they ask I also do the test. When I went I was also positive, no he(husband) is negative, we came here and they did it for him twice after they requested I ask him to come. He agreed to come with me and he came and did it. For me and him child is only God who knows how we got this sickness.

Yes, I was coming for hospital here (TTH), when I was pregnant for ANC. Yes, they(facility) did and it was negative, I wasn’t having it and when I delivered I was negative. She was about three years that I realized the child was positive after she started falling sick frequently. Where were u admitted TTH here at the children ward. I was counseled and they gave me the request to do the lab and the doctor spoke to me after the results were positive and directed us here to see mama

And we came here she spoke to me well, and they gave them folder and I started the medications

I was surprised but I leaved everything to god, I was surprised and shocked but I did not cry and was calm. He (husband) just kept quiet and he said he does not know where this sickness came from.

**Can you tell me about how you seek care and support?**

She was growing lean; I always come to the hospital, no one support but is my husband that Yes whenever we are sick and need money for test or medicine that they write and they don’t have it at the hospital he provides. Yes, they (my family members) come, sometime they come with food and other things.

Me I don’t have friends, yes they (neighbors) do visit when am on admission, (my assembly man, Imam and community leaders), No please. For the support Something better could be done but we are appreciative of what they do for us.

**What are the roles and activities they take in the care of x?**

When I wake up I will let her go to look for water to bath, then I will give her food and send her to school before I go to work. Her madams usually bring her from school. She takes it in the evening for the disease medicine and multivitamin in the morning. When she takes the medicine in the evening she always feels dizzy so she sleeps.

**Emotional, spiritual and physical support/coping/relax**

I always sleep, and most often pray and make recitations of the Quran. But I don’t like watching television, I don’t have that time but my child does.

**Disclosure**

Nobody knows of our condition aside my husband because we know this condition you can easily; they will spread it to everybody. No I don’t want to tell any body

**Tell me about the care and expectation from the hospital**

They always take care of our weight, blood pressure before they give us our medicine, they take good care of us and make sure were are comfortable. They take good care of us (when on admission) until she recovers fully before they will discharge us. We need food stuff to make the child eat well and grow better. My problem also is the thing, am praying for God that the thing, they get the cure to the sickness to take it away especially the children. And also if they can get us dome money to care for the children, especially the school fee.

**Can you tell me about home and hospital medications for x? Perceived barriers to enrollment and side effects of ART.**

Is not all of us who are the same, some of them feel shy to bring the children, there some others who naturally do not like the hospital medication and will not bring the children for it. Some people don’t want people to know their sick of this condition for them to make fun of them. Because here if people just hear something small they will just spread it

She feels dizzy and like she is dozing, in the evening they go the mosque to recite the Quran, when she takes it and go she feel sleepy. She will not sit up two hours before going into sleep. Then she will come and say Mama am feeling sleepy. Sometimes she coughs, and does not complain of abdominal or bodily pains.

**What is the most challenging issue/ day in caring for x?**

The problem is many people disturb me and ask me why it is that she like falling sick our neighbors

**What kind of discriminations do you encounter due to x condition?**

No please because many people don’t know she is with this condition

**What are your plans for x in the future**?

If pray they get the cure and she will be free. I pray she continue to be independent and go about her normal duties

**What else do you want to tell me?**

I always ask for the injection to cure the disease. But am told the cure is not in but will come.

**Caregiver** 8: TWH o1, 31/10/ 19,

**Can you tell me about yourself?**

I use to be a trader but has for some time now do not work again as my business has failed, so for now I don’t do any work. We are 12 people, my husband has two wives, he is a Mallam. Aside that of being a Mallam does he do any work. We stay alone as a nuclear family but we go to the family house often which is closer, the head of the family is my father in-law, the bread winner is my husband but he is not gainfully employed as he does religious activities so money becomes a problem. Even my mother too does not work, am supposed to help her but now I don’t have money, first that I use to work I help her small small she is an old lady. No one says anything am the one who determines what to do and bring him to the hospital to take medicine, we don’t have money if they write medicine for me, so we don’t go because we don’t have money. So we just collect the paper and keep it. Sometimes he is the one that brings him to the hospital he helps a lot. We (and the extended) are good together but I personally do not have anybody that helps me much

**Can you tell me about x? Talk about the girl, how you got to know about her condition**

Since birth I have been caring for him, and he has since been diagnosed 7 years ago and I have been caring for him. I have four children and they were all good but during my last pregnancy for the younger sister of this boy, I come to the hospital and I was told I was positive. When I got to the house i planned to kill myself. Because I was thinking only me how can I carter for the child and myself, so i bought poison to drink, I kept the poison under my bed and my sister in- law saw it and ask me severally why I kept those smelling poison under the bed, I confided in her and inform her of the diagnosis when I came to the hospital. She intends telling my husband. Hmmmmm he is a good man he consoled me and ask me and told me not to worry much is the making of Allah. He also got tested two consecutive times and was negative.

I started the medications and gave birth to my girl ehhmm the last born. But after some time I realized the boy was falling sick often and affected his growth and schooling since he did not go to school often. I decided to bring him to this unit to be tested for the virus. To my surprise he was positive so I have to tell my husband, Alhaji (head of art unit), we not also belief so ask Alhaji write the test for us to do at TCH. Hmmm we did not believe the results. The father sent him there the following Monday and he was tested and they brought the results here and it was positive. That is how I got to know the status. My husband said is ok everything is God

Yes, I was not told (I had the virus), is the current girl that I got to know. It hurt me so much, when I got home I cried seriously. I willingly brought him for the test when I realized I was positive and how the child was suffering. so he was kept on medications by Alhaji. So we don’t take herbal medicine as my husband said herbal medicine cannot help with that sickness. So is only this medicine that we always collect for him

**Can you tell me about how you seek care and support?**

We always send him to the hospital and we pray that you get the cure to the disease.

No no no we don’t get any support in caring for the child. I don’t have anyone except my husband. Sometimes you don’t know how to tell the person, you may tell the person, the person will hear and will not help you. When he gets money small he will give to me for cooking, yes and when he is going to school. He does not have money; I can’t disturb him if he does not have. Some husbands when they hear that their wives have the disease they, they will say they don’t know you and sack you, for him he has done well. So I don’t want to disturb him again. I know of a man who sacks the wife because she has the disease. For my husband he has done well, initially he thought he also has the disease but he does not have. When am sick they (neighbors) come to visit me and pray for, sometimes they bring food but they don’t offer money. They just come and visit and go home. Since I have been coming on admission none of them has offered me even one cedi. No please they (assembly man and Imam) don’t come. If you have an Imam who does not come to see, then you don’t have one (laughing). The help is not that good but we are managing because for human what you have is what you have to eat and manage. Or am lying.

**What are the roles and activities they take in the care of x?**

After morning prayers, I wake them up, sweep the compound, bath for them particularly e this boy because of how he is, cook for them or prepare tea or sometimes I don’t have money to buy milk to prepare their tea, I used the small money to buy koko for them, to go to school, days I have money I give each of them especially this one two cadis each, and days I don’t have I talk to them and promise them a meal after school for them to eat when they return l, hmmm sometimes when they come and complain the meal is not sweet I beg them to eat and become satisfy , they come at 3 00 pm I wash their clothes every day, I don’t want them to wear dirty clothes , then the same for the evening since I don’t have work. I give his medicine in the evening around 6: 00 pm when we are going to sleep, then we pray. I give it in the evening because I know he will not go out again. Because after he has finish taking it he becomes drowsy. Even mine too is in the evening that I take mine.

But if I forget I give him immediately the next day after bathing and taking tea I will give him. If it happens that way that morning, I have to pray to get money to give him much money to buy food at school. The burden is severe, for this boy everyday he is sick he complains of headache and stomach pains, I spend a lot on him for medications, sometimes I don’t have money to buy medicine and even send him to hospital. Because sometimes the hospital people become feared up, every day same complain, and they and will tell you is the same medicine they will give you. My husband last even said if he had money he will send him to private hospital for them to see him well. Because he complains of these a lot and he can’t tell what actually is wrong with him

**Emotional, spiritual and physical support/coping/relax**

If I were working it would have help or if I had some money to continue my business it will help me and the boy paah, you know when you don’t have work and you sit down it will not help, that gives me a lot of thinking. Hmmmm at first I was big paah, now I don’t work everyone will go out and only me will be in the room, you can’t even sleep, even in the night I can’t sleep will ly down like that that aaahh I will not be sleeping, I will be thinking. If you look at the disease it demands, you eat well meanwhile you don’t have. But I do pray, I pray to Allah to get me a helper if your family will not help, I guess god can use a new person to help u because is not only your family member alone that can help you, and watch television. I don’t have friends to chart with because they can cause problems.

**Disclosure**

Is only my husband and sister in –law who knows. But I wanted to tell my mother but I can’t tell her. My husband said she is an old lady we should not tell her because it will hurt her. She does not have anything she is very very old and I don’t want to tell her. I really wish to tell her, first I wanted to do it and my husband refuses. (friends and neighbors) No no please I will not tell them

Because you can’t tell who is good or am lying, yes, for some now you are no problem but tomorrow when you quarrel they will use it to insult you. Then your everything will be basaah then disgrace will come. So when you keep it a secret is better while you pray to god that the cure is found. In the night I can pray like that aaaa when I can’t sleep. Even if you tell them they will not help you and for all the family I don’t wants to tell any of them

**Tell me about the care and expectation from the hospital**

The care is good, they do well if I say anything bad I will be lying. Even when my date comes and I don’t come and I come they understand me and give me my medications

But at the lab not this current man ooo but the previous one when he sees me he will talk. When I was pregnant with this boy and he saw me he said ehhiii you are pregnant again, and j was embarrassed. What he said was not good because he is not god, then I asked him is there anything the matter, when I did that and shouted at him he said when I deliver I should call him he will give the child some medicine. And I told him I will not call him. Hmmm I don’t really know the medicine. Yes, I did tell him (Alhaji / the unit head) and he told me not to mind him, because first Alhaji said he will call him and talk to him that what he doing is not good. It makes people hurt and uncomfortable. Because what he was doing was as if what is going on can kill me now, now now is not good. Even if you know that you have to console the person, because everything is the making of god. Because there are people without this condition and are dying.

I have even told Alhaji that they should try and help us because we are not employed. Sometimes when I see how the child is it makes me sorrowful. I expect foodstuff, money for supporting us

**Can you tell me about home and hospital medications for x? Perceived barriers to enrollment and side effects of ART.**

Yes, some of them die for not taking the medicine, I even know someone she uses to bring the child here for medicine so I know her but she stops coming, then one dying I was passing by her store and I saw her child and I asked her why she has not been bring the child for medicine. She then sends me outside and ask me why I was asking and I told her am positive and my son too and we go for the medicine. If am telling you to go for it, it’s not a crime. And I told her I did not say it in public its private between us and as I see the child this was not how sick the child is so send her to the hospital and take the medicine and for them to check the child is good. She did not talk to me again, because she has her business and she is doing so it does not border her .as am telling you the child has died long time curahhh

Some care giver believes those who say they have the cure to the disease so they go there a they don’t come here. That is what is preventing most of the people. Others too when they are coming they think they know some people who may see them. Sometimes the money for transport is a problem sometimes. For me sometimes I hold my bag and walk here with him. Then I will go to the school and time them he is sick and I brought him to hospital. They will understand and allow me to send him home to take the medicine then the next day he will come, but some don’t want to do that. I can even come here when they have not come and I will sit here and wait for them. Am very punctual for the medication if they are going to award I will be first. Sometimes when I come here and see some sorrowful client I talk to them and inform them I also have it.

Yes, many of them, but I don’t believe and my husband. I don’t know how someone can convince me to take such herbal medicine. Herbal medicine can’t cure this disease

Sometime he overheard they said they have done a research and has had the cure he came to me to inform him he had heard a good news told me this is what he heard and I also told him I have heard about it on the TV. Then he said lets pray for those trying to help get the cure.

He feels dizzy, especially when he is not well feed, he gets weak small, at first he uses to cough but know nothing of that sort. No ooo I can’t tell, but all the time that’s his complain (headache and stomach pains), then I become worried because I don’t know what will happen to him

**What is the most challenging issue/ day in caring for x?**

Is that he falls sick often it gives me thinking, sometime ago he felt sick seriously in the nigh, this body was so warm, and when you see his eyes it was reddish I managed through the night and early in the morning I bathed him ago he and I brought him to this hospital and they admitted him for three days he was given water (infusion) and air before he got better and we were discharged. We spent money paahhh. I remember one day at school he felt sick seriously and his madams brought him home to me, they were asking me a lot of questions why almost all the time he is falling sick like that and they said when they teach he understands faster so when it gets to sometime s and he absent from school frequently is not good. And I told them I have heard them and they leave. They were concern for the boy but it did not mean I should tell them. It disturbed me, because he is good at school and the disease is disturbing her and I don’t know what to do. If money were there I will seek better care for him, now that there is no money what will I do.

**What kind of discriminations do you encounter due to x condition?**

I don’t really ask him of those things because if I hear them it will hurt me, especially at school so I don’t ask

**What are your plans for x in the future**?

He wants to be a doctor and I pray he becomes, all the times that’s what he says.

**What else do you want to tell me?**

Anything that can be help to live with this, because we don’t have money, sometimes food, and since with money we can get food we need their help that and it can help us set up business to supplement us. Because even when we get borrow to start business and it fail how do we go with it and they will come asking of the money it will increase our problems with the disease. So finally I will say they should help us to help us care for the children. If they help us their gift will be with God hands. Because we don’t have a family member that will help us and if someone helps that person becomes a family. Because taking the medicine is good but without food it will not help that much and you can’t help you. And the medicine too the supply sometimes may not be plenty so instead of collecting two or three months you may be given one which disturb with the person taking the medicine.

**Caregiver** 9: TWH 02

**Can you tell me about yourself?**

Please I wash clothes, the clothes that I was when the sickness kept me down I laid down for I year. So when I wake up they said my strength has gone down small so I should not do hard work again, I should not have left heavy things like water again I should go near fire again then it happened that way when I was at coming here (Tamale from Nalerigu). My house people didn’t agree because if I come here and I don’t do heavy work I will not be able to take care of myself and the child. I don’t also have anybody that will help me here like that. I don’t do any other work. We are three, myself and my two children but the other one school at GHANASCO stay together here Shishagu. He (husband) is a Mayson but he does secondary school work as laborer. I lives in an extended family but currently I stay in Tamale here alone in a nuclear family. (head of family) my husband own or my own. For my husband house I left there for long, when the disease came out the man left me. Myself is the clothes I wash that I use to care for the child

(Family cohesion) Ehhiii when it came this way the man said it will come out that this disease is on me, so I should hide about the disease but my brothers told me if I do that it will kill me. So when it happened that way he sacked me from the house, then the child was known to have the virus. He said his father says I should go with the child. he said am a big funeral and the child is a small funeral, so we should go. But they should collect the elder brother who is negative, so I left there. If either of us is sick is on me, we do go to hospital but because I don’t usually have money we go to church for prayers after before we go to hospital but I really would have want to go Kumasi for prayers. When the sickness started on me we use to take herbal medicine but when we were enrolled onto the ART drugs we were held not to take herbal medicine.

**Can you tell me about x? Talk about the girl, how you got to know about her condition**

What happened was that it seems when I was pregnant that the child had the disease or when I gave birth the child breastfeeding for long that’s why. During those times I did not that disease was o me. The child sucked for 3 years. When she was sick, it was looked like malaria, so when it happened she for three days her face changed and we sent her to the hospital. By then I was with my husband at Nalerigu and I sent her that it looked like she was going. after they saw her they then asked us to see one doctor called Sisu. By then I was taking the ART so the doctor then told me is the same but the sickness that was on the child. So I should stop thinking and collect the medicine for the child, I knew I were positive the sickness kept me down for one year and I got well before the child got sick by then the child was about 3 or 4 years. I was seriously sick for long, my body spoiled and my brothers are many at Nalerigu, so they encouraged me to go there and check and I did that is how I got to know am sick. The truth is I made the sickness come out, there was a fight and I travelled to Accra. So when I returned I went to my father’s place. My brothers advised I went to my husband place.

But while at my father’s house people told me if am to go back to my husband place I should be careful because when I was away people have been saying my husband is sick with that disease. So when they said that it touched my heart, if is like that I will check myself and if is true and am living with the condition I will go to my husband and if I don’t have it we will stop (divorce), so the next day I went to the hospital so when I went and the doctor asked me and I said that, he said am I sure and I said yes.

I just want to check, they asked me several questions and I told them I came so they should do it for me. if is like that I will take the medicine and know what to do. When they checked it was true I was positive. that’s how I got to know my status.So the doctors together with my pastors called my husband to inform him he did not agree to come. They tried severally but he never came. So when I got home I could not eat, every day I was crying I was thinking plenty. It got to sometimes that he agreed to take the medicine but refused to do the test. And the people refused to do that. I was still staying with him. I began falling sick seriously but he was not taking care of me again, then my family said if he does not take care of me they have to bring me home. He was not in agreement so there were fights so my people took me home by force and god willing I got well by one-year time. His father said I have disgraced them that I have allow people to know am living with that disease. So I stayed at my father’s house for long then I came to Tamale to stay for almost 3 years’ now. The (news) It disturbed me because the child when it happened I knew she was innocent and is because of me all this happened so who will take care of us since the father was not supportive. I was thinking the father will be considerate and let care for ourselves well. But he followed his father to treat us bad. So I was guilty knowing the child too is sick because if were to be me only it would have been better than the child too.

After I was counseled that day when the news of the child being positive was done my husband and my brothers quarreled. And he slapped my brother and beat him severely, latter he thought my brother was dying so he choked himself with a knife to cover up if there were to be an arrest. so I was rushed to my father’s house. My husband took the elder one who is not positive and asked they should bring the small funeral to me at my father’s house. since they can’t care for her because they said if I die it will not be long before she will also die. Hmmm I was not comfortable because we don’t use money to buy sickness, the doctor did well and comforted me. When I was pregnant I went to the hospital, I did they did several test but I was not told I was positive my first two children are negative.

**Can you tell me about how you seek care and support?**

I bath her and buy her some medicine at the pharmacy or bring her to the hospital. And I usually bring her to the unit here and they will usually ask me to take a folder for her at the OPD. Hmmm they care for us well our last time we were admitted for the about 10 days and I was glad with how they took care of her. The truth is hmmm because the way the disease is many people are afraid of the disease. The family does not help when am sick or the girl none of them give me even five cedi’s or ten cedi’s to buy medicine. If we are sick and I don’t have money, we lie down like that. if my Health Insurance dies and we are sick it disturbs us. Since I travelled here five years ago none of my family members knows where I am. I don’t have friends do you refer to boyfriends, because I don’t have a partner I have tried severally and failed, but is one of my co-tenants a lady but we are not friends, when on admission no one comes to visit. I have one child at GHANASCO all the expenses are on me, the father does not care.

Here these Daghomba they don’t really help. And because it is an urban area each one for himself but when am sick and they don’t see me out for days my neighbors ask my child or myself. for instance, last Saturday I was seriously sick and did not come out. Today while I was coming out one of them asked me she has not seen me for some time. So they come to greet but giving something no and they do pray for me as well. My pastor is married to my family lady so she comes to greet us and sometimes bring us food but for the pastor he himself no. When am sick he does not come home to pray for me unless I go to the church to request for prayers. For assembly man, no I don’t even know him, is good for human if he comes to greet you and does not even give you anything is good. I am appreciative because most of these people are not my direct family members is nice to me. The father does not offer any financial help but these days he can call and ask to talk to the girl. Sometimes when he calls I will say I will not pick but my brother advised me to pick. some pastors help their congregants especially the widows but in my case not like that

**What are the roles and activities they take in the care of x?**

when I wake up in the morning I will set fire, I will wake her up to go and fetch water and she baths, then we take tea then she dresses and go to school. I will be in the house and wait If anybody will bring clothes, I will wash, if not I have to be staying indoors. If I get I will wash and the person will come and take them in the evening. In the evening I will prepare food. I don’t want her to walk around so if she comes we will be indoors like that. She will read her books small and we will watch TV small. Is someone who bought us the TV so that the child will not have to roam about. In the evening sometimes she will tell me to take a walk. then she sleeps and the next day again. In the evening she takes her medicine. I remember three years ago she did something at this unit and everybody was crying. She was then small she said she was going home this medicine that ewe collect all the time, since she was small she has taken this medicine like that aaahhh from the water one to the tablets. I told her it was malaria and she said no so she said since she was a child and the malaria is not going? she will not take the medicine again. So when she said that I cried. I thought she will not take the medicine again but when is time she takes hers and remove mine for me with water. She is able to do all things, I can send her, but I don’t want to force her that much to do work much. She plays with other children but I don’t want her to stay outside for more than 6 pm

**Emotional, spiritual and physical support/coping/relax**

Coping is difficult but I manage, I think a lot I can wash clothes plenty and when I am squatting my waist will be paining me. As if I should cry because I could think far paaahh that is not me it because a major problem. But when it happens that way it looks like I think of suicide but the child will suffer if I die so I pray to god that he knows and gave us this disease. He should support us as always. I do chart with the woman in the house especially in the evening, if not her sometimes I can sleep in the room like that aaahhhh, sometimes too I walk around with the child to the roadside if I have a coin I will take it then we will go and come. Use the coin to buy something outside

**Disclosure**

ahhhh hmmm bro hmm in this world, one of my sister who is the pastors’ wife and the pastor knows here in tamale, but for my hometown Nalerigu, everybody knows that is more why I travelled here to live. And most of them know is from the man, and they always pray I should recover and be alive. When I was sick seriously and be able to care for my child, for others here I have not told because they will spread the news of me and my girl being sick. Is difficult to tell others but my family I told all of them. My co-tenant in my house if I tell her she will run away from the house, when I came new she uses to help us a lot. It did not take long for us to be friends. When am sick she could cook and the next morning make tea for me in the evening she could prepare fufu for me. She uses to do very well even if is medicine she buys for me. even as I speak I have abdominal pain I bought medicine but yesterday he gave me medicine as well she takes care of me well but if I tell her she will run away.

**Tell me about the care and expectation from the hospital**

They take very good care of us, if we come they don’t maltreat us, when I come with her they play with her very nicely. then she becomes happy, they never say anything that disturbs us. Some medicine like septrin first they use to give us especially for abdominal pain but now they don’t give, because it good psss if u take it, it helps a lot but we are asked to buy.at the drug store yourself. We ladies we need to care for the child, I did not go to school and I don’t have a job, sometimes it may be one week, two weeks or 3 but I have not had work. Then it becomes a problem if we could be helped to get work to get money to care for yourself and the children or some money to start small business like sell ice water to support ourselves, and food for instance if I don’t eat no problem but for the child if she does not eat taking the medicine will be a problem so we want food. If you don’t eat and you take the medicine it will do you as if you are drank, so thus the major concern because we dont do work, because am told my heart has a problem it makes me want to go home but me want to do a check up to see if my strength is better. First they use to give us maize oil among others when this comes we use to be happy because if help us. Because we will not buy that again is only fish and salt we will have to buy. I don’t belong to any association but I think is good I want a partner and I think with such I will get one. You see when you are two it helps if have the worries are many, some people can still flirt even in this condition but I don’t want to do that. Where I stay in Nalerigu if u try with any of the men, there it will not take long for the information to spread to him. But with such organization u can get someone positive and you will all know and live together it is good that way. They could come from different places Kumasi, Accra etc for us to meet it use to give us hope.

**Can you tell me about home and hospital medications for x? Perceived barriers to enrollment and side effects of ART.**

Some of them I don’t know if they don’t know what is wrong with them for me after lying down for a year I know what is wrong with me and my girl.

If you do that u have disturbed the child because you gave birth to the child into this world and this happened to the child. to me those who do that is not good. some don’t believe the disease is true. Some where they stay is far for them to come and take the medications, some soon has not informed their family for them to support and may be coming to the hospital every month, others will ask why their coming like that and because they don’t want to disclose they will soon stop coming. Bodily weakness, one day we were sleeping and because they had changed the medicines to tablets she wake up in the night sleeps wake up and sleep again. Then I asked her Catherine why are you doing that then she said something is doing her that if she tells me I will beat her that is as if I have drunk alcohol. Meanwhile I also feel so then she said since they changed the tablets. She does not vomit though

**What is the most challenging issue/ day in caring for x?**

Sometimes I cry because I have three children why did it come on this poor girl.it can hurt me paaahh and the child will be asking me questions, when she see me in such state she does not go out again

**What kind of discriminations do you encounter due to x condition?**

God has blessed me on that since it started and I took medications for her since has not been sick often again she has been admitted on twice. When she is sick is her head or stomach not so serious. If she goes to school and she is not well the teachers will ask her to go home for them to take her to hospital and if she and if she is well then she will come. There is no discrimination as such. Because people don’t know her status and whenever she is not well I go to the school to tell them.

**What are your plans for x in the future**?

To able to go to school and get work to live independently.

**What else do you want to tell me?**

We need help plenty, the child goes to school and we need help because the father had has disserted us if she happens to further at SSS it will be difficult for me.

Thank you for your time
