# Supplementary material for: “Some believe those who say they can cure it” perceived barriers to antiretroviral therapy for children living with HIV/AIDS: Qualitative exploration of caregivers experiences in tamale metropolis
Source: PLoS One. 2022 Oct 4;17(10):e0275529. doi: 10.1371/journal.pone.0275529 (PMC9531795; doi:10.1371/journal.pone.0275529)
Supplement: Attachment — Submitted filename: Plos 1 Review 29 Nov.docx [file pone.0275529.s003.docx]

Review

Overall

- Great study, very interesting findings. I think the part about herbal / traditional is very important in the Ghanian context, so I found that particularly interesting.
- Some of the sentences could be restructured to be more concise or clear. For example, the minutes of interviews were 47, this would be better structured as “The interviews lasted an average of X minutes.” which is more active and clearer. I would recommend doing a read outloud to find small structure issues like this.
- I would like to understand better what is unique about this study compared to other similar studies. I would like a better understanding of the context and population and how this study makes a unique contribution. This is not quite clear to me now, but there is much potential here. I think expanding on Tamale and the population would help.
- Did religion come up at all? I know Northern Ghana is largely muslim so I wondered if any sort of religious factors arose?

Abstract

- This is minor but there are some typos in the abstract which take away from the quality of the work. For example, “there exist” should be “there exists”.
- “HIV and AIDS” is later referred to as “HIV/AID”. I would make this consistent throughout. The abstract is a bit long/unbalanced-- the background is very long whereas the results are short.

Ethics Statement

- Do not need to say the second “respectively”.

Title

- I would remove the capitalizations from Cure and Believe in the title, as it is a quote. It would potentially be better to be consistent about capitalization in the whole title.

Introduction

- Pick HIV and AIDS rather than saying it that way sometimes, and other times using HIV/AIDS. I would recommend the latter.
- Some citations are missing in the first few sentences of the introduction. For example, for “as many more infected are having their quality and expectancy of life improving.” “diseases of history have exerted systematic and structural effects”.
- My same comment about sentence structure is relevant in the introduction. For example, the sentence “Evidence from Ghana shows CLWHA (0–14 years) are about 25,955(8%) with an estimated 2,972(15%) new infections and 2,441 (18%) deaths.” should be something more like, “In Ghana, there are 25,955 0-14 year olds (8%)...” to make it more active and clear.

Methods

- “Fathered by Husserl the meaning and essence people have about a phenomenon is described while making sure presuppositions are bracketed(25)” I don’t understand this sentence and generally the first paragraph. Can it be explained in a less jargony fashion?
- First sentence about Tamale is missing a citation
- Which hospitals were selected? Name them
- The sentence “The hospitals which serve as training centers for students, provides in-patient and out-patient care services” should be re-phrased. “The hospital has a training center for student, inpatient care services, and outpatient care services.”
- “Their exposition of the phenomena perceived barriers because they were asked, what are some of the reasons that prevent other caregivers from bringing their CLWHA to the ART clinics for treatment?” Is this the only question that was asked? Could you include the questionnaire with the paper? This sentence seems out of place given the Instrument Section exists.
- The steps of Colaizi could be better presented in a figure or a chart of some kind rather than in a dense paragraph.
- Add citations for Colaizi and Lincoln and Guba
- Don't understand this sentence please rephrase “Haven obtained ethical clearance the hospital management and ART clinic in charges were notified before data collection began”

Results

- Good summary of participants!
- I like the presentation of themes

Discussion

- These sentences are missing something in the beginning, I guess an author name: “(15) identified inability to buy food, the burden of taking multiple medications and school attendance limiting privacy as barriers in Uganda among adolescence living with HIV/AIDS which contrast this current study” “33) corroborates the position of this current study when it was noted that the mere presence of a person at the HIV counselling clinic is enough for the person to be labelled as or suspected to be an HIV patient. Indicating a high perception of stigmatization which serve as a barrier to ART.”
- Some of the studies it is compared to could be summarized, rather than a separate sentence about all of them
- The last paragraph before strengths and limitations could be expanded, especially if the other literature is summarized a bit more concisely. I would expand on these recommendations and consider grounding them in other studies which have shown these suggestions have been successful elsewhere.
