# Supplementary material for: “Some believe those who say they can cure it” perceived barriers to antiretroviral therapy for children living with HIV/AIDS: Qualitative exploration of caregivers experiences in tamale metropolis
Source: PLoS One. 2022 Oct 4;17(10):e0275529. doi: 10.1371/journal.pone.0275529 (PMC9531795; doi:10.1371/journal.pone.0275529)
Supplement: Attachment — Submitted filename: Reviewer response..docx [file pone.0275529.s004.docx]

Tamale Central Hospital,

Pediatric Department,

Post Office Box TL2649,

Ghana,

Northern Region,

Tamale.

16^th^ Jan., 2022

The Editor,

PLOS ONE Journal,

Dear Sir,

**REVIEWERS RESPONSE LETTER FOR RESEARCH ARTICLE TITLED**

**“SOME BELIEVE THOSE WHO SAY THEY CAN CURE IT” PERCEIVED BARRIERS TO ANTIRETROVIRAL THERAPY FOR CHILDREN LIVING WITH HIV/AIDS: QUALITATIVE EXPLORATION OF CAREGIVERS EXPERIENCES IN TAMALE METROPOLIS**

Following reception of reviewers comment of the above, the authors provides the understated responses.

| **Comment** | **Page** | **Reviewer response** |
| --- | --- | --- |
| **Reviewer 1** | | |
| On page 9, the authors state, ‘Owing to the varied biographic background (age, sex, and religion) of the caregivers which did not favour focused group discussion’, as a reason for opting for in-depth interviews. However, focus group discussions (FGDs) can also be categorised according to the different participants’ characteristics to achieve a homogeneous group for a balanced discussion. By itself, this may not be a justifiable reason for choosing in-depth interviews over FGDs, and the authors should clarify if there were any other reasons besides that | 9 | Comment acknowledged and further explanations provided |
| Could the authors give more details about how data saturation was determined. Was iteration considered in the data collection and analysis to guide this process? | 9 | Yes, iteration was considered and we have provided further information on saturation. |
| The term ‘HIV positive’ is generally considered stigmatizing and ‘people living with HIV’ is preferred. The authors should edit this throughout the manuscript | Throughout the script | Comment acknowledged and corrections made |
| From the illustrative quotes presented, stock-outs and privacy at the clinic relate to two different barriers. Why did the authors choose to organise these under the same theme? | 16 | We recognized both barriers as related to facility/hospital level barriers.  As such put them together |
| On page 15, under theme two, the authors report, ‘Aside one of the sites which has a separate ART clinic away from the facility main structures, the other sites ART clinic had privacy issues. In one it was located at the main Out-Patient Department (OPD) and the other at Ante-Natal Clinic (ANC) closer to the hospital administration.’ Was this reported by the caregivers or was it an assessment made by the authors through observation? Data collector observation may not necessarily reflect the perceptions of the caregivers and is not covered under the stated data collection methods. | 15 | Comment acknowledged however, caregivers narrated how the proximity of the clinics to OPD and administration made it easy for others to identify them as PLWHA during our final step of data validation (member checks)  The introduction of adding such information is to make it much clearer for readers. |
| On page 21, Paragraph 1, the authors make the conclusion that because confidentiality and poor coordination of testing and treatment were not identified as barriers in this study, unlike findings in previous studies, ‘there exist properly trained counsellors and HIV professional providing services’ and that ‘This contrast underpins better professional ethics and conduct of care providers in this metropolis.’ However, this assumption may not hold and it is not supported by findings of the study. | 21 | Upon a careful consideration, this portion has been deleted from the manuscript. |
| In paragraph 2, the authors also write that, ‘Substance abuse was not mentioned in this study as a barrier because a majority of the caregivers were females.’ It may be useful to explain how gender is related to alcohol use and present supporting references. |  | The relationship between gender and alcohol abuse has been explained and referenced. |
| The authors pose sample size as a limitation of the study. However, if sampling was done until data saturation was reached, as reported, this ought not to be a concern. How do the authors think having a larger sample size would have improved the study? | 25 | We accept the comment and do, delete same, Thank you |
| Wouldn’t it have been useful to also include caregivers of children who were not on ART to explore barriers to ART enrolment? The authors could consider including this as a limitation of the study. | 25 | Suggestion accepted and added to the limitations |
| In the last statement, the authors conclude that, the caregivers ‘are able to navigate these challenges due to resilience and need to ensure their CLWHA are healthy’. This however does not seem derived from the study findings. | 25 | The nature of these perceived barriers shows these caregivers equally faces same. Its therefore their resolve to adhere to medications that makes them navigate these barriers |
| Some references for statistics cited are not provided for example in the last paragraph on Page 4 and first paragraph on page 5. | 4 and 5 | The said references have been provided. |
| Were the caregivers required to speak both English and Daghani or either of the two. Please clarify on Page 8. | 8 | They were required to speak either of the two.  Amends made |
| On page 9, was the presence of a witness during the consenting process a requirement for all participants, including those who could read and write? | 9 | Yes, please it was required for all |
| It may be better to write in full female and male, instead of F and M, or give a key at the bottom of the table | 14 | Comment accepted and corrections made |
| Unemployed may be a better term to use than ‘Nill’ under occupation | 14 | Comment accepted and corrections made |
| It would be good to specify what the ‘other’ under ‘relationship to the child’ is for. | 14 | Comment accepted and corrections made |
| The authors in the first paragraph on Page 12 write that, ‘three (3) [of the caregivers] involved in self-employment (laundry, seamstress and petty trading) which does not provide regular income’. Was the conclusion that these occupations do not provide regular income made by the authors based on the nature of the work or was this self-reported by the caregivers? | 12 | Please it was self-reported |
| Some of the referencing needs formatting, for example, in the first sentence in paragraph 1 on page 22. | 22 | Reference formatted as suggested |
| Some statements are incomplete, for example, the second last paragraph on page 22, ‘Active involvement of significant others in clinic appointment by care providers.’ Generally, language editing is recommended for the entire manuscript to improve readability. | 22 | Comment acknowledged  Sentence completed |
| **Reviewer 2** | | |
| Some of the sentences could be restructured to be more concise or clear. For example, the minutes of interviews were 47, this would be better structured as “The interviews lasted an average of X minutes.” which is more active and clearer. I would recommend doing a read outloud to find small structure issues like this. | Throughout the script | Language and other editing has been made throughout the entire script |
| I would like to understand better what is unique about this study compared to other similar studies. |  | Our study is unique compared to others, because it explored barriers from caregivers who had enrolled their children on ART and were adhering to appointments. Thus enquiring from them why other caregivers were not enrolling and/or adhering to appointment.  This is taken from the context that in ghana ART coverage is 46.55% among adults (15+ years), 53.76% women (15+ years) and just 25.55% children (0-14years).  For children living with HIV/AIDS their caregivers are the most critical people in their lives since they are dependent.  Our study also contrast other studies that explores barriers to ART among adults |
| I would like a better understanding of the context and population and how this study makes a unique contribution |  | Context is taken from the premise that in Ghana ART coverage is 46.55% among adults (15+ years), 53.76% women (15+ years) and just 25.55% children (0-14years).  As such nearly a seventh of children living with HIV/AIDS are not enrolled unto ART compared to a much better statistic for adults and women.  Yet, the power of making this very important decision lies in the hands of adults.  To this end since ability to recruit caregivers of children living with HIV/AIDS not enrolled and /or adherent was difficult, we approached caregivers of adherent children. This was to explore their “perceived” reasons why other caregivers refuse to enroll their children and /or adhere to appointments.  The findings provide unique knowledge about barriers to ART which are also confronted by adherent caregivers. Thereby providing provident solutions that are useful for both categories of caregivers in the metropolis. |
| This is not quite clear to me now, but there is much potential here. I think expanding on Tamale and the population would help. |  | Indeed, we recognize so  But in this study we purposively selected facilities with ART clinics for this study. |
| Did religion come up at all? I know Northern Ghana is largely muslim so I wondered if any sort of religious factors arose? |  | Yes, it did come up  But was not strongly attached to any of the faiths as reviewer sought to know |
| This is minor but there are some typos in the abstract which take away from the quality of the work. For example, “there exist” should be “there exists”. “HIV and AIDS” is later referred to as “HIV/AID”. I would make this consistent throughout. The abstract is a bit long/unbalanced-- the background is very long whereas the results are short. |  | Thank you for your comment  Amends has been made |
| Do not need to say the second “respectively”. | 13 | Comment acknowledged and amends made |
| I would remove the capitalizations from Cure and Believe in the title, as it is a quote. It would potentially be better to be consistent about capitalization in the whole title. | 1 | Comment acknowledged and corrections made |
| Pick HIV and AIDS rather than saying it that way sometimes, and other times using HIV/AIDS. I would recommend the latter. | Throughout the script | Comment acknowledged and amends made |
| Some citations are missing in the first few sentences of the introduction. For example, for “as many more infected are having their quality and expectancy of life improving.” “diseases of history have exerted systematic and structural effects”. | 5 | References has been provided |
| My same comment about sentence structure is relevant in the introduction. For example, the sentence “Evidence from Ghana shows CLWHA (0–14 years) are about 25,955(8%) with an estimated 2,972(15%) new infections and 2,441 (18%) deaths.” should be something more like, “In Ghana, there are 25,955 0-14 year olds (8%)...” to make it more active and clear. | 3 | Sentence has been reframed as suggested |
| Fathered by Husserl the meaning and essence people have about a phenomenon is described while making sure presuppositions are bracketed(25)” I don’t understand this sentence and generally the first paragraph. Can it be explained in a less jargony fashion? | 7 | Comment acknowledged and amends made |
| First sentence about Tamale is missing a citation | 7 | Reference provided |
| Which hospitals were selected? Name them | 7 | Amends made and hospitals named |
| The sentence “The hospitals which serve as training centers for students, provides in-patient and out-patient care services” should be re-phrased. “The hospital has a training center for student, inpatient care services, and outpatient care services.” | 7 | Comment acknowledged and amends made |
| “Their exposition of the phenomena perceived barriers because they were asked, what are some of the reasons that prevent other caregivers from bringing their CLWHA to the ART clinics for treatment?” Is this the only question that was asked? Could you include the questionnaire with the paper? This sentence seems out of place given the Instrument Section exists. | 9 | The interview guide has been included  And some of the probes added to the text |
| The steps of Colaizi could be better presented in a figure or a chart of some kind rather than in a dense paragraph. | 12 | Comment acknowledged and the sentences has been put into a figure (1) |
| Add citations for Colaizi and Lincoln and Guba | 12 | Reference provided |
| Don't understand this sentence please rephrase “Haven obtained ethical clearance the hospital management and ART clinic in charges were notified before data collection began” | 13 | Comment acknowledged and amends made |
| These sentences are missing something in the beginning, I guess an author name: “(15) identified inability to buy food, the burden of taking multiple medications and school attendance limiting privacy as barriers in Uganda among adolescence living with HIV/AIDS which contrast this current study” “33) corroborates the position of this current study when it was noted that the mere presence of a person at the HIV counselling clinic is enough for the person to be labelled as or suspected to be an HIV patient. Indicating a high perception of stigmatization which serve as a barrier to ART.” Some of the studies it is compared to could be summarized, rather than a separate sentence about all of them | 23 | Comments acknowledged and corrections made |
| The last paragraph before strengths and limitations could be expanded, especially if the other literature is summarized a bit more concisely. | 25 | Comment acknowledged and amends made |
| I would expand on these recommendations and consider grounding them in other studies which have shown these suggestions have been successful elsewhere. | 1 | The section has been reviewed and more recommendations added base on literature |

Changes on financial disclosure should be as stated below

The study did not receive any external funding but personal contributions from the authors.

Is our hope this meet your kind consideration and expedite action.

Thank you

Yours sincerely

Gideon Awenabisa Atanuriba

[atanuriba@gmail.com](mailto:atanuriba@gmail.com)

+233541186103
